# *Lactobacillus fermentum *(PCC^®^) supplementation and gastrointestinal and respiratory-tract illness symptoms: a randomised control trial in athletes

**DOI:** 10.1186/1475-2891-10-30

**Published:** 2011-04-11

**Authors:** Nicholas P West, David B Pyne, Allan W Cripps, William G Hopkins, Dorte C Eskesen, Ashok Jairath, Claus T Christophersen, Michael A Conlon, Peter A Fricker

**Affiliations:** 1 Department of Physiology, Australian Institute of Sport, Canberra, 2617, Australia; 2 School of Physiotherapy and Exercise Science, Griffith Health, Griffith University, 4222, Australia; 3 School of Medicine, Australian National University, Canberra, 0200, Australia; 4 School of Medicine, Griffith Health Institute, Griffith University, 4222, Australia; 5 AUT University, Auckland, 1142, New Zealand; 6 Chr. Hansen A/S, Hørsholm, Denmark; 7 Probiomics Ltd, Australia, 2072, Australia; 8 CSIRO, Human Nutrition, Adelaide, 5000, Australia; 9 Executive, Australian Institute of Sport, Canberra, 2617, Australia

## Abstract

**Background:**

Probiotics purportedly reduce symptoms of gastrointestinal and upper respiratory-tract illness by modulating commensal microflora. Preventing and reducing symptoms of respiratory and gastrointestinal illness are the primary reason that dietary supplementation with probiotics are becoming increasingly popular with healthy active individuals. There is a paucity of data regarding the effectiveness of probiotics in this cohort. The aim of this study was to evaluate the effectiveness of a probiotic on faecal microbiology, self-reported illness symptoms and immunity in healthy well trained individuals.

**Methods:**

Competitive cyclists (64 males and 35 females; age 35 ± 9 and 36 ± 9 y, VO_2_max 56 ± 6 and 52 ± 6 ml.kg^-1^.min^-1^, mean ± SD) were randomised to either probiotic (minimum 1 × 10^9 ^*Lactobacillus fermentum *(PCC^®^) per day) or placebo treatment for 11 weeks in a double-blind, randomised, controlled trial. The outcome measures were faecal *L. fermentum *counts, self-reported symptoms of illness and serum cytokines.

**Results:**

*Lactobacillus *numbers increased 7.7-fold (90% confidence limits 2.1- to 28-fold) more in males on the probiotic, while there was an unclear 2.2-fold (0.2- to 18-fold) increase in females taking the probiotic. The number and duration of mild gastrointestinal symptoms were ~2-fold greater in the probiotic group. However, there was a substantial 0.7 (0.2 to 1.2) of a scale step reduction in the severity of gastrointestinal illness at the mean training load in males, which became more pronounced as training load increased. The load (duration×severity) of lower respiratory illness symptoms was less by a factor of 0.31 (99%CI; 0.07 to 0.96) in males taking the probiotic compared with placebo but increased by a factor of 2.2 (0.41 to 27) in females. Differences in use of cold and flu medication mirrored these symptoms. The observed effects on URTI had too much uncertainty for a decisive outcome. There were clear reductions in the magnitude of acute exercise-induced changes in some cytokines.

**Conclusion:**

*L. fermentum *may be a useful nutritional adjunct for healthy exercising males. However, uncertainty in the effects of supplementation on URTI and on symptoms in females needs to be resolved.

**Trial registration:**

The trial was registered in the Australia and New Zealand Clinical Trials Registry (ACTRN12611000006943).

## Background

Probiotics are becoming increasingly popular as a nutrition supplement to reduce susceptibility to common infectious illnesses, particularly upper respiratory tract (URT) and gastrointestinal (GI) illness. For athletes, reducing the occurrence of these illnesses is a high priority. There is a heightened incidence of URT and GI illness, particularly diarrhoea, during heavy training and competitions [1-3]. The increased susceptibility to illness is thought to relate to acute post-exercise immune perturbations and chronic suppression of immune factors due to frequent heavy exercise [4,5]. These perturbations in immunity are proposed to provide a window of opportunity for micro-organisms to establish infection. Illness during heavy training or competition may have negative consequences for athletic performance [6]. As such, probiotics, may offer a nutrition strategy to limit illness affecting performance.

There is increasing evidence that probiotic supplementation, alone or in combination with other preventative agents such as prebiotics, can reduce the number, duration and severity of acute infectious diarrhoea and URTI in the general population [7-9] and in at risk sub-groups, such as the elderly [10]. This evidence has led to interest in the use of probiotics to reduce illness in athletes. Moderating or ameliorating acute and chronic exercise-induced perturbations in immunity via the use of probiotic supplementation may be one strategy of reducing susceptibility to illness. Initial research examining the efficacy of probiotics in physically active groups has thus far been inconclusive. Three studies indicated that probiotic supplementation might be useful for enhancing immunity and reducing the duration of URTI and GI illness in endurance-based athletes [11-13], whereas probiotic supplementation by commando cadets during a training and combat course had little effect on the incidence of URTI [14]. These findings are not surprising given sample size, length of the supplementation and illness observation period and differences in probiotic strain and dosage [15]. Despite the mixed findings from scientific research, however, probiotics are increasing in popularity in healthy active groups, mirroring the trend generally in developed countries [16]. Given the inconsistent findings of efficacy between physically active groups and the growing popularity of probiotic supplements, it is necessary that the benefits of supplementation are substantiated to ensure individuals make informed choices.

Concerns on the safety and efficacy of probiotics have recently led to calls for the development of interpretive frameworks on the ratio of clinical benefit to risk [17]. In most published probiotic studies (and other dietary supplements) knowledge of the risks has not been considered when characterising the potential benefits of use. Analytical approaches that specify clinically important thresholds of effect, either positive or negative [18], rather than only statistically significance, offer better clinical insights for making decisions regarding the use of probiotics as a supplement.

The primary aim of this study was to determine the effects of supplementation with *Lactobacillus fermentum (PCC^®^) *on URTI and GI symptoms in a cohort of healthy, physically active individuals over a 15-week winter training period. A secondary aim was to establish the effect of supplementation with *L. fermentum (PCC^®^) *on faecal microbiology and key aspects of immunity at rest and in response to an exercise test to exhaustion.

## Methods and Subjects

### Study design

The study involved a randomised, double-blind, placebo-controlled parallel-groups design consisting of an initial 2-wk pre-supplement (baseline) period, 11.0 ± 0.4 wk (mean ± SD) of supplementation and a 2-wk post-supplement follow-up period. Subjects were recruited and randomly allocated using a computer generated list to either probiotic or placebo treatment based on gender, age and maximal oxygen uptake (VO_2_max) by a researcher independent from the study team. Participants and the study team were blinded to the interventions until the completion of the statistical analysis as the probiotic and placebo were manufactured to be identical in packaging, encapsulation and taste.

Cyclists and triathletes from Canberra, Australia and its surrounding regions were contacted via email alerts and during competition to participate in the study by the main researcher. One hundred and nine cyclists volunteered. Subjects were required to declare their use of other dietary and/or ergogenic aids that may have influenced underlying immune function and/or exercise performance. All subjects on immuno-modulatory medications were excluded. Inclusion in the study was dependent on the subjects not taking antibiotics or supplements/foods containing probiotics for at least one month prior to and during the study period. Subjects were also required to have a maximal oxygen uptake (VO_2_max) of at least 45 ml/kg/min for women and 50 ml/kg/min for men. Ninety-nine subjects met the study requirements.

Subjects consumed one capsule daily of either the probiotic or placebo supplement. The probiotic capsule contained a minimum of one billion (10^9^) colony-forming units of *Lactobacillus fermentum *VRI-003 PCC^® ^(Probiomics Ltd, Sydney, Australia). This dose was chosen on the basis of commercial viability and is consistent with other probiotic studies showing efficacy for URTI and GI illness [19]. The placebo supplement consisted of microcrystalline cellulose. Subjects were able to consume the supplement at any time with or without food. Subjects returned the bottles following supplementation and capsules were counted to verify the degree of compliance. All subjects completed a four day food diary during the study that incorporated two week days and a weekend to allow adjustment for the effect of dietary food intake on microflora. Verbal and written guidelines were provided to subjects to ensure foods were recorded accurately. Detailed descriptions including brand name, packaging, method of preparation and quantity were recorded. Subjects were asked to maintain a normal diet beyond the instruction to refrain from eating probiotic-enriched yoghurt and probiotic or prebiotic enriched foods or supplements. All records were reviewed by a dietician. Total energy (kJ), carbohydrate (g), fat (g), protein (g) and fibre (g) were assessed by using FoodWorks professional edition software package (version 3.0, Xyris Software, Brisbane, Australia).

This study was conducted according to the guidelines of the Declaration of Helsinki and all procedures involving human subjects were approved by the Human Research Ethics Committees of the Australian Institute of Sport and Griffith University. All participants provided written informed consent.

### Illness symptoms

Subjects recorded symptoms of GI, URT and lower respiratory illness on a daily illness log over the study period, as previously described [20]. Briefly, symptoms of GI illness included nausea, vomiting, diarrhoea, abdominal pain, abdominal bloating, flatulence, stomach "rumbles" and loss of appetite. URTI symptoms included throat soreness, sneezing, a blocked or runny nose and cough. Lower respiratory illness symptoms included coughing with chest congestion and/or wheezing. Two or more symptoms on at least two consecutive days were defined during the analysis as an episode of illness. Symptoms separated by only one day were counted as the same episode. The severity of symptoms were self-rated as mild, moderate or severe based on the impact of the symptoms on training for that day, with mild symptoms resulting in no change to training, moderate symptoms necessitating a reduction in training volume and/or intensity, and severe symptoms leading to a total cessation of training on that day[20]. The duration and mean severity of each episode were calculated. Subjects were also asked to record all medications consumed during the study, including antibiotics, anti-inflammatories, pain killers, decongestants and anti-histamines. Dietary supplements that might have influenced underlying immune function or exercise performance were also recorded.

### Training and performance measures

Subjects recorded information on all types of physical activity undertaken during the study. For each training session subjects recorded training distance (km), duration (min) and intensity (scored on a 1-5 scale: 1, easy; 5, maximal). At the start and end of the study subjects undertook an incremental performance test to determine peak power output, VO_2_max and the acute post-exercise cytokine response. The test was performed on a cycle ergometer (Excalibur Sport, Lode NV Groningen, the Netherlands) as previously described [21].

### Sample collection

Saliva and blood samples from all subjects, and faecal samples from a cohort of 20 subjects from each group (10 male and 10 female), were obtained pre- and post-supplementation. Saliva was collected using an eye spear (Defries Industries, Victoria, Australia). The eye spear was placed between the cheek and teeth for up to five minutes, centrifuged for 5 minutes at ~800 g and frozen at -80°C until analysis. Albumin concentration and osmolality was assessed to control for changes in salivary flow rate and hydration. All saliva samples were taken at the same time of the day to control for diurnal variation. Blood samples were taken prior to and immediately after the acute exercise challenge undertaken at the start and end of supplementation. Pre-exercise post-prandial samples were used to determine whether supplementation enhanced resting plasma cytokine concentrations. Blood samples taken immediately after the exercise challenge were used to determine if supplementation ameliorated the acute post-exercise plasma cytokine response. Blood samples were drawn directly into K_3_EDTA tubes (Greiner Bio-one; Frickenhausen, Germany) from an antecubital vein immediately before and after the exercise test to exhaustion. Plasma was separated by centrifugation at ~800 g for 5 min and stored frozen at -80°C until analysis. Faecal samples were collected in a sealable plastic bag and frozen immediately in a portable -20°C freezer (Waeco Pacific Pty Ltd).

### Measures of mucosal immunity

Lactoferrin, lysozyme and SIgA concentrations were measured spectrophotometrically by enzyme linked immunosorbant assay (ELISA) using commercial kits (lactoferrin - EMD Chemicals, New Jersey, USA, lysozyme - Sapphire Bioscience Redfern Australia, SIgA - Salimetrics, IgA -Salimetrics, Philadelphia, USA). Albumin concentration was measured by immunoturbidimetric assay on a Hitachi 911 Chemistry Analyser (Roche). Osmolality was measured on a Model 3320 Osmometer (Advanced Instruments Inc) as per the manufacturer's instructions. The inter-assay variability for high and low controls in the lactoferrin, lysozyme and SIgA assays are included in Table three. Variability was acceptable at < 10% for the low and high positive controls.

### Measures of systemic immunity

The cytokines analysed were granulocyte macrophage-colony stimulating factor, (GM-CSF), interleukin (IL)-1RA, IL-6, IL-8, IL-10, tumor necrosis factor (TNF)-α and interferon gamma (INF-γ). The concentration of plasma cytokines were measured on a Bio-Plex Suspension Array System (Bio-Rad Laboratories Pty Ltd; Hercules, CA, USA). The samples were analysed on custom manufactured Multiplex Cytokine Kits (Bio-Rad Laboratories Pty Ltd; Hercules, CA, USA). Plates were read using the Bio-Plex Suspension Array System (Bio-Rad Laboratories Pty Ltd; Hercules, CA, USA). A full blood count including white cell count and differential were analysed on a haematology analyser (Advia, GMI, MI, USA). Results from each assay were accepted if the positive controls were within two standard deviations from their established mean concentration. The CVs for the low and high controls ranged from 4.5% to 12%.

### Molecular microbiology

DNA was extracted according to Abell et al. [22] and quantified using Quant-iT™ Picogreen (Invitrogen). Microbiome diversity was examined using a universal bacteria 16S rRNA primer set (907f - 1392rgc). The amplified product was analysed by denaturing gradient gel electrophoresis (DGGE). The PCR and DGGE gel conditions followed the protocol of Abell et al. [22] with the exception that a 35% - 70% denaturing gradient was used. Dominant DGGE bands were extracted from the gels and sequenced for putative identification. DNA was extracted from the DGGE bands using a modification of the method described by Boom *et al. *[23]. The DGGE bands were excised from the gels using a x-tracta (Geneworks, Hindmarsh, SA, AU) and transferred to a 1.7 ml tube and submerged in 100 μl of diffusion buffer (0.5M ammonium acetate; 10 mM magnesium acetate; 1 mM EDTA pH 8.0; 0.1% SDS) followed by incubation at 50°C for 30 min. The extracted DNA was then re-amplified using the primers 907f and 1392r without the GC-clamp and sequenced in both directions by capillary separation on an AB 3730*xl *sequencer. The sequences were then quality checked and assembled using the software packed ChromasPro version 1.41 (Technelysium Pty Ltd). The complete sequences were then putatively identified using Basic Local Alignment Search Tool (BLAST) [24] and GenBank. DGGE banding patterns were analysed to estimate bacterial diversity for each specimen using GelCompar II version 6.0 (Applied Maths, Inc., Texas, USA) software package and the normalised banding patterns were further analysed with Primer6 version 6.1.12 and Permanova+ addition version 1.02 (PRIMER-E Ltd, Plymouth, UK)[25].

Quantitative real-time PCRs (QPCR) were performed in reaction volumes of 20 μl containing 1X IQ™ SYBR^® ^Green Supermix (Bio-Rad Laboratories, Hercules, CA) and 0.2 mg/ml BSA using a Chromo-4 thermocycler (Bio-Rad Laboratories, Hercules, CA). Results were analysed with the Opticon Monitor 3 software (ver. 3.1) (Bio-Rad Laboratories, Hercules, CA). All assays were performed in duplicates with primer pair specific thermocycler programs followed by melt curve analysis to assure specificity of primers. The program for detection of the total bacterial population [26] consisted of an initial 4 min, 95°C hot start followed by 35 cycles of 95°C for 20 s, 60°C for 20 s and 72°C for 45 s with fluorescent acquisition after each cycle. The *Lactobacillus*[27,28] and the *Bifidobacterium*[29] group was detected using the following parameters, 95°C hot start for 4 min then 35 cycles of 95°C for 20 s, 58°C for 20 s, 72°C for 30 s and 80°C for 30 s followed by acquisition. The *Clostridium coccoides spp*. [30] and *Bacteroides fragilis *[31] assays had identical parameters, 95°C hot start for 4 min then 35 cycles of 95°C for 20 s, 58°C for 20 s and 72°C for 30 s followed by acquisition. The PCR parameters for the detection of *Escherichia coli*[32] were as follows: 94°C hot start for 4 min followed by 35 cycles of 94°C for 30 s, 20 s at 60°C and 45 s at 72°C with fluorescent acquisition after each cycle.

### Statistical analysis

Our analytical approach centred on the practical/clinical significance of probiotic supplementation rather than statistical significance alone. We made inferences about the true (large-sample) effect of the supplement on a given symptom based on the uncertainty in the effect in relation to the smallest clinically important values [33,34]. A full description of the approach utilized is available in additional file 1.

Descriptive statistics of all measures are presented as mean ± standard deviation. The effects of supplementation on illness symptoms were more precise for the post-only analysis (rather than traditional pre-post analysis) and are reported here. All symptoms were analysed per 100 days and a proportion of each subject's symptom scores in the shoulder periods was assigned to the subject's accumulated scores to account for the start of supplementation and the monitoring period following the end of supplementation. The number of symptom episodes of a given symptom per 100 days, total number of days of the symptom per 100 days, and total load of the symptom per 100 days (sum of the product of symptom severity and number of days of the symptom per 100 days) were analysed as ratios: the mean of the probiotic group divided by the mean of the placebo group during treatment. Ratios of 1.20 and 1/1.20 ( = 0.83) were chosen as smallest clinically important differences. Effects on symptom severity and on all training-related measures were analysed as differences rather than ratios of means. Pre-specified sub-group analysis included examining the data by gender, age and training load. The smallest important effects for these measures were derived by standardisation: 0.20 of the pooled between-subject standard deviation in the control group and probiotic groups [35]. Differences between group means of subject characteristics were assessed with a modification [33] of Cohen's scale [35] for standardised effects (small, 0.20-0.60; moderate, 0.60-1.20; large, > 1.20). Confidence limits for the effects on symptom scores and training measures were obtained with bootstrapping. These analyses were performed with the Statistical Analysis System (Version 9.1, SAS Institute, Cary, NC).

Effects of the probiotic on measures of immunology and enteric microflora (Q-PCR data) are presented for pre-post analyses. These measures were log-transformed before analysis to permit the effect of the treatment to be properly analyzed as factors or percents, and magnitudes of effects were determined by standardisation of the log-transformed variable. We used the t statistic for independent samples with unequal variances. Baseline values of the dependent variable were included as a covariate in these analyses to account for regression to the mean. We also investigated the extent to which bacterial counts accounted for symptom scores in this subsample of subjects. In these analyses the log-transformed bacterial count or the pre-post change in the log-transformed count was the covariate, and the dependent variable was rank-transformed.

A sample size of 80 subjects was required for identifying substantial changes in the incidence of illness [20]. We assumed a rate of upper respiratory tract illness symptoms of 60% in the placebo group, with sufficient power (86% at an alpha-level of 0.05) to detect a 50% reduction in symptoms.

## Results

### Subjects

A total of 99 subjects were initially allocated to treatment groups but only 88 completed the 11 weeks of supplementation. One subject dropped out after moving interstate while the remaining subjects did not complete the self reported training data and were subsequently excluded from analysis. There were no substantial differences between the treatment groups in the time spent training per week, the number of exercise sessions per week, session intensity or mean weekly training load or dietary intake. Subjects were physically and physiologically similar (Table 1). Dietary intakes were considered low for an athletic population but not abnormal [36]. Body mass of the athletes did not change substantially over the course of the study (data not shown). Supplementation with *L. fermentum (PCC^®^) *had no substantial effect on the training patterns or performance in post-testing VO_2 _max testing of athletes in the experimental group over the duration of the treatment period (data not shown). The consort checklist can be found in additional file 1.

**Table 1:** Physical and physiological characteristics of the subjects

	Probiotic	Placebo	Qualitative difference
**Males**			
N	29	33	
Age (y)	35.2 ± 10.3	36.4 ± 8.9	Trivial
Mass (kg)	77.9 ± 8.4	76.9 ± 8.2	Trivial
VO_2_max (ml/kg/min)	56.5 ± 6.2	55.8 ± 5.6	Trivial
RBC (×10^12^/L)	5.0 ± 0.3	5.1 ± 0.4	Small
WBC (×10^12^/L)	6.2 ± 1.7	6.3 ± 1.6	Trivial
Energy intake (kJ)	8954 ± 2870	9138 ± 3168	Trivial
Fibre (g)	16.4 ± 6.9	19.3 ± 11.8	Trivial
Lactobacillus CFU/g	1.4 × 10^04 ^± 2.5 × 10^04^	3.8 × 10^06 ^± 1.3 × 10^07^	Large
**Females**			
N	18	17	
Age (y)	36.5 ± 8.6	35.6 ± 10.2	Trivial
Mass (kg)	57.6 ± 6.1	61.3 ± 9.9	Small
VO_2_max (ml/kg/min)	53.0 ± 5.0	51.6 ± 7.4	Small
RBC (×10^12^/L)	4.7 ± 0.2	4.7 ± 0.3	Trivial
WBC (×10^12^/L)	5.2 ± 1.0	7.6 ± 1.7	Large
Energy intake (kJ)	7491 ± 1336	8133 ± 2512	Trivial
Fibre (g)	15.5 ± 5.1	18.6 ± 14.1	Trivial
Lactobacillus CFU/g	1.4 × 10^04 ^± 9.4 × 10^04^	1.8 × 10^04 ^± 1.7 × 10^04^	Trivial

VO_2_max, maximal oxygen uptake; RBC, Data are mean ± SD. red blood cells; WBC, white blood cells; CFU, colony forming units

### Symptoms of illness

The patterns of self-reported gastrointestinal illness symptoms in probiotic and placebo groups over the treatment period are shown in Table 2. For male and female subjects taking *L. fermentum (PCC^®^) *there was a two-fold increase in the number and duration of mild (low-grade) self-reported gastrointestinal symptoms. No substantial effect of supplementation was evident between the groups in moderate and severe GI symptoms. However, the self-reported severity score for GI illnesses at the mean training load in males on the probiotic was 0.7 (0.2 to 1.2) of a scale step lower than for males on the placebo, and this positive effect of the probiotic increased with higher training loads. The effects of probiotic supplementation on URT illness load were unclear in males (0.66; 0.23 to 1.78, mean, 99% confidence intervals) and females (0.99; 0.23 to 3.37). Effects of supplementation on other measures of URTI were also unclear. In male subjects, self-reported symptoms of lower respiratory illness (i.e. number, duration and severity) were ~50% lower in the probiotic group (Table 3). In contrast, we observed a two-fold increase in the number and duration, but a reduced severity, of self-reported symptoms of lower respiratory illness of symptoms in females taking *L. fermentum (PCC^®^)*. The effects of probiotics on measures of medication usage (Table 4) generally mirrored the effects on lower respiratory illness. Covariate analysis in the sub-sample that provided a faecal sample did not reveal clear trends between patterns of illness symptoms and supplement-induced changes in gut bacteria.

**Table 2:** The effect of probiotic treatment on the number, duration, severity and combined load of gastrointestinal symptoms

			Effect of probiotic treatment relative to placebo (mean; 99% CI)		
				
	Probiotic group (mean ± SD)	Placebo group (mean ± SD)	Ratio	Difference	Qualitative inference
**Males**					
Number of episodes	1.01 ± 1.70	0.49 ± 0.90	2.06; 0.51 to 11		Likely ↑
Duration (d)	3.3 ± 5.8	1.3 ± 7.7	2.57: 0.53 to 17		Likely ↑
Severity (1-3 scale)	1.31 ± 0.33	1.78 ± 0.73		-0.47; -1.21 to 0.28	Possible ↓
Symptom Load (severity-days)	4.4 ± 8.1	2.5 ± 5.8	1.76; 0.34 to 15		Possible ↑
**Females**					
Number of episodes	1.44 ± 1.71	0.48 ± 0.71	3.02; 0.76 to 17		Likely ↑
Duration (d)	3.9 ± 5.4	2.1 ± 4.3	1.85; 0.35 to 27		Possible ↑
Severity (1-3 scale)	1.44 ± 0.68	1.75 ± 0.88		-0.31; -1.39 to 0.79	Possible ↓
Symptom Load (severity-days)	5.2 ± 7.08	2.9 ± 5.1	1.81; 0.37 to 24		Possible ↑

SD, standard deviation; CI, confidence interval.

Smallest clinically important values for assessing effects qualitatively: ratios, ×/÷1.20; differences, ± 0.5

**Table 3:** The effect of probiotic treatment on the number, duration, severity and combined load of lower respiratory symptoms

			Effect of probiotic relative to placebo (mean; 99% CI)		
	Probiotic group (mean ± SD)	Placebo group (mean ± SD)	Ratio	Difference	Qualitative inference
**Males**					
Number of episodes	0.6 ± 0.9	1.0 ± 1.5	0.55; 0.15 to 1.60		Likely ↓
Duration (d)	3.5 ± 6.6	7.4 ± 10.3	0.47; 0.10 to 1.43		Likely ↓
Severity (1-3 scale)	1.23 ± 0.36	1.65 ± 0.57		-0.43 -0.92 to 0.08	Possible ↓
Symptom Load (severity-days)	4.0 ± 7.2	13.0 ± 19.0	0.31; 0.07 to 0.96		Very likely ↓
**Females**					
Number of episodes	1.6 ± 2.1	0.5 ± 0.8	3.27; 0.82 to 21		Likely ↑
Duration (d)	12.2 ± 14.8	5.1 ± 14.7	2.39; 0.45 to 43		Likely ↑
Severity (1-3 scale)	1.38 ± 0.45	2.07 ± 0.77		-0.69 -1.57 to 0.10	Possible ↓
Symptom Load (severity-days)	17.1 ± 19.0	7.9 ± 20.7	2.18; 0.41 to 27.23		Likely ↑

SD, standard deviation; CI, confidence interval.

Smallest clinically important values for assessing effects qualitatively: ratios, ×/÷1.20; differences, ± 0.5.

**Table 4:** The effect of probiotic treatment on the number of, duration of use, severity and combined load of medications usage

			Effect of probiotic relative to placebo (mean; 99% CI)		
			
	Probiotic group (mean ± SD)	Placebo group (mean ± SD)	Ratio	Difference	Qualitative inference
**Males**					
Number of medication episodes	0.37 ± 0.72	1.26 ± 1.90	0.29; 0.04 to 0.89		Very likely ↓
Total days of medications	5.0 ± 20.8	7.2 ± 14.8	0.70; 0.01 to 2.3		Unclear
Mean # of medications per episode	1.14 ± 0.24	1.29 ± 0.44		-0.15; -0.57 to 0.24	Possibly trivial
Total # of medications	5.2 ± 20.8	9.1 ± 17.3	0.57; 0.01 to 3.1		Unclear
**Females**					
Number of medication episodes	1.98 ± 2.63	1.08 ± 1.32	1.83; 0.38 to 6.5		Possible ↑
Total days of medications	10.7 ± 18.2	5.8 ± 7.9	1.86; 0.31 to 8.5		Likely ↑
Mean # of medications per episode	1.15 ± 0.24	1.53 ± 0.99		-0.39; -1.32 to 0.24	Possible ↓
Total # of medications	12.7 ± 20.9	7.4 ± 10.4	1.70; 0.27 to 7.8		Possible ↑

SD, standard deviation; CI, confidence interval.

Smallest clinically important values for assessing effects qualitatively: ratios, ×/÷1.20; differences, ± 0.5.

### Systemic immunity

Table 5 shows that supplementing with *L. fermentum (PCC^®^) *attenuated acute exercise-induced changes in both anti- and pro-inflammatory cytokines (IL-1RA, IL-6, IL-8, IL-10, GM-CSF, IFN-γ, TNF-α) in males and females adjusted for mean training load. There were ~20-75% smaller perturbations in anti-inflammatory and pro-inflammatory cytokines in males and females taking the probiotic following supplementation. There were no substantial differences between the treatment groups in resting cytokine concentrations from pre- to post-supplementation. We failed to identify substantial relationships between the reduced exercise-induced perturbations in cytokines and illness symptoms after supplementation. The full data on the cytokine concentrations can be found in the additional files (Additional file 1, Tables S1 to S4).

**Table 5:** Effect of probiotics on acute post exercise cytokine response (factor changes)

	Probiotic group (mean ×/÷ SD)	Placebo group (mean ± SD)	Ratio effect of probiotic treatment relative to placebo (mean; 99% CI)	Qualitative inference
**Males**				
Anti-inflammatory				
IL-1ra	0.84 ×/÷ 2.42	1.39 ×/÷ 1.42	0.60; 0.41 to 0.88	Very Likely ↓
IL-10	0.95 ×/÷ 1.69	1.16 ×/÷ 1.44	0.78: 0.61 to 0.99	Possible ↓
Immuno-regulatory				
IL-6	0.92 ×/÷ 2.14	1.22 ×/÷ 2.1	0.75; 0.51 to 1.1	Likely ↓
Pro-inflammatory				
IL-8	0.80 ×/÷2.46	0.87 ×/÷ 2.02	0.71; 0.60 to 1.25	Unclear
GM-CSF	0.78 ×/÷ 2.66	1.75 ×/÷ 2.20	0.45; 0.28 to 0.72	Very Likely ↓
IFN-γ	1.2 ×/÷ 2.30	1.49 ×/÷ 2.10	0.81; 0.54 to 1.21	Likely ↓
TNF-α	1.27 ×/÷ 2.02	1.66 ×/÷ 2.22	0.77; 0.51 to 1.14	Likely ↓
**Females**				
Anti-inflammatory				
IL-1ra	0.80 ×/÷ 2.44	1.88 ×/÷ 2.16	0.42; 0.23 to 0.78	Very Likely ↓
IL-10	0.89 ×/÷ 1.84	1.45 ×/÷ 2.03	0.61; 0.38 to 1.0	Possible ↓
Immuno-regulatory				
IL-6	0.71 ×/÷ 2.93	2.29 ×/÷ 4.8	0.31; 0.11 to 0.84	Likely ↓
Pro-inflammatory				
IL-8	0.71 ×/÷ 2.7	1.15 ×/÷ 2.5	0.62; 0.30 to 1.27	Probably ↓
GM-CSF	0.85 ×/÷ 2.75	3.3 ×/÷ 3.9	0.25; 0.10 to 0.64	Very Likely ↓
IFN-γ	1.07 ×/÷ 2.8	1.56 ×/÷ 2.1	0.68; 0.47 to 1.0	Likely ↓
TNF-α	1.15 ×/÷ 2.0	1.72 ×/÷ 1.8	0.67 0.47 to 0.94	likely ↓

SD, factor standard deviation; CI, confidence interval.

Smallest clinically important values for assessing effects qualitatively: ratios, × ÷1.20; differences, ± 0.5.

### Mucosal immunity

There were no substantial differences in the concentration of the mucosal immune measures lactoferrin, lysozyme or SIgA between the probiotic and placebo groups from pre- to post-supplementation (data not shown). The concentrations of all proteins in saliva were characterised by large within- and between-subject variability (coefficients of variation ~200-400%). Further data can be found in additional file 1, Tables S5 to S6.

### Faecal microbiology

Q-PCR analysis revealed a moderate 330% increase (90% confidence limits; 50% to 1170%) in males in the probiotic group and a small 44% decrease (-76 to 27%) in males in the placebo group in total *Lactobacillus *numbers from pre to post supplementation. Taken together these changes underpinned a 7.7-fold difference (2.1-fold to 28-fold) in total *Lactobacillus *numbers between the groups. There were trivial differences in total *Lactobacillus *numbers between females, with those supplementing with the probiotic having a 6-fold (0.7-fold to 47-fold) increase and those in the placebo group having a 3-fold (1.3-fold to 6-fold). There were no substantial differences in the abundance of *E.coli, C. coccoides, Bifidobacterium *and *Bacteroides fragilis *group between treatments. Raw bacterial counts are detailed in Table 6.

**Table 6:** Raw bacterial counts at pre- and post-supplementation

	Probiotic group Pre (mean ± SD)	Probiotic group Post (mean ± SD)	Placebo group Pre (mean ± SD)	Placebo group Post (mean ± SD)
**Males**				
Total bacteria	2.5 × 10^10 ^± 1.9 × 10^10^	2.0 × 10^10 ^± 9.0 × 10^9^	2.2 ×10^10 ^± 1.2 × 10^10^	1.7 × 10^10 ^± 1.0 × 10^10^
C. coccoides	1.1 × 10^09 ^± 1.4 × 10^09^	8.7 × 10^08 ^± 1.2 × 10^09^	1.2 × 10^09 ^± 1.2 × 10^09^	8.6 × 10^08 ^± 6.7 × 10^08^
E. coli	1.6 × 10^05 ^± 1.5 × 10^05^	8.0 × 10^05 ^± 1.4 × 10^06^	2.4 × 10^05 ^± 4.5 × 10^05^	9.2 × 10^05 ^± 2.5 × 10^06^
Bifibacteria	1.7 × 10^07 ^± 2.4 × 10^07^	1.4 × 10^07 ^± 1.6 × 10^07^	1.0 × 10^07 ^± 1.8 × 10^07^	4.4 × 10^06 ^± 8.8 × 10^06^
Bacteroides	2.3 × 10^06 ^± 5.0 × 10^06^	2.9 × 10^06 ^± 5.2 × 10^06^	2.1 × 10^06 ^± 2.0 × 10^06^	3.7 × 10^06 ^± 6.7 × 10^06^
Lactobacillus	1.4 × 10^04 ^± 2.5 × 10^04^	7.2 × 10^04 ^± 1.3 × 10^05^	3.8 × 10^06 ^± 1.3 × 10^07^	1.0 × 10^06 ^± 3.5 × 10^06^
**Females**				
Total bacteria	1.9 × 10^10 ^± 8.3 × 10^10^	2.6 × 10^10 ^± 1.9 × 10^10^	3.6 × 10^10 ^± 2.2 × 10^10^	2.6 × 10^10 ^± 2.7 × 10^10^
C.coccoides	1.0 × 10^09 ^± 1.1 × 10^09^	1.0 × 10^09 ^± 8.9 × 10^08^	2.5 × 10^09 ^± 2.9 × 10^09^	9.6 × 10^08 ^± 8.6 × 10^08^
E. coli	6.4 × 10^06 ^± 1.5 × 10^07^	2.0 × 10^07 ^± 4.8 × 10^07^	7.6 × 10^03 ^± 8.0 × 10^03^	5.1 × 10^04 ^± 7.2 × 10^04^
Bifidobacteria	2.9 × 10^06 ^± 2.9 × 10^06^	3.6 × 10^06 ^± 5.1 × 10^06^	7.4 × 10^06 ^± 1.2 × 10^07^	1.3 × 10^06 ^± 1.3 × 10^06^
Bacterioides	1.2 × 10^06 ^± 1.2 × 10^06^	2.5 × 10^06 ^± 3.9 × 10^06^	5.1 × 10^07 ^± 9.9 × 10^07^	6.6 × 10^06 ^± 9.6 × 10^06^
Lactobacillus	1.4 × 10^04 ^± 9.4 × 10^04^	8.4 × 10^04 ^± 7.8 × 10^04^	1.8 × 10^04 ^± 1.7 × 10^04^	8.7 × 10^04 ^± 1.4 × 10^05^

## Discussion

Supplementation with *L. fermentum *PCC^® ^was associated with a reduction of symptoms in clinical indices of lower respiratory illness, GI symptoms at high training loads, and cold and flu medication use in well-trained male cyclists. These outcomes were not evident in well-trained females, with some evidence of an increase in symptoms. An increase in mild GI symptoms most likely reflects an adaptive response of the GI tract to alteration in the composition of the microflora. The increased recovery of total *Lactobacillus *species in faeces may have underpinned the clinical outcomes. Collectively these studies indicate that *L. fermentum (PCC^®^) *may be a useful nutritional adjunct for physically active males in both competitive and recreational settings.

The favourable clinical findings of this study are consistent with previous work undertaken by our group examining *L. fermentum (PCC^®^) *in highly trained runners [12]. A reduction in severity of illness with probiotic supplementation should enable individuals to maintain daily activities and, in the case of athletes, training and competitive performance. Episodes of illness often occur during heavy exercise training periods [6], a time when athletes obtain the greatest improvements in fitness [37]. Illness that interrupts individual training sessions may prevent athletes from maximising the effects of their training program. The reduction in GI symptom intensity at higher training loads has implications for multi-day events and competitions. These events are typically characterised by high volume, high intensity exercise and occasionally an increase in GI symptoms [38]. The finding that supplementation reduced symptom severity offers a potential intervention for these athletes.

The explanation for the higher number and duration of self-reported symptoms of lower respiratory illness in females supplementing with the probiotic is unclear and difficult to reconcile with the reduced severity of symptoms. Clinical and immunological differences between the sexes are well recognised [39]. It is possible that these divergent clinical findings were an artefact of sampling variation, given the large number of analyses reported in the study. However, taking the male and female results together, the findings with the symptoms are consistent with changes in cold and flu medication usage. Further work is required to clarify this apparent discrepancy between the sexes in physiological and clinical responses to probiotic supplementation.

We were unable to identify an effect of probiotic supplementation on URTI in contrast to a previous study in our laboratory [12]. The effect of the probiotic may have been more pronounced had there been a higher rate of illness. The individuals recruited to this study were well-trained, competitive athletes undertaking daily exercise. Some individuals undertaking prolonged intense exercise regularly may suffer a higher rate of illness than moderately trained individuals [40]. The subjects in this study may not have been as active as the earlier study, where the elite cyclists and triathletes in that study typically expended four times more energy on a daily basis than recreationally active individuals similar to our subjects.

An increase in mild GI symptoms in individuals taking the probiotic is consistent with other research examining probiotic supplementation in healthy individuals [19]. Mild transient abdominal bloating, flatulence and cramping have been reported by subjects during the initial stages of probiotic supplementation [19]. These symptoms are thought to reflect adaptive changes in the gastrointestinal tract in response to the introduction of additional bacterial species, and a subsequent alternation in fermentation activity. In our study, however, symptoms did not abate after the initial 7-10 days and remained higher in number in the probiotic group throughout the period of supplementation. The mild GI symptoms in this study did not affect an individual's ability to undertake exercise training and are of little functional (athletic) consequence. Similar to other clinical probiotic studies [41], we found that despite a higher number and duration of GI symptoms in those supplementing with *L. fermentum (PCC^®^) *the severity of symptoms was lower. This reduction in severity can be easily explained: the increase in the number and duration of mild symptoms dilutes the overall symptom severity.

The increased susceptibility to illness associated with exercise may relate to exercise-induced perturbations in immunity [42], which provide a window of opportunity for infection. A previous study by our group found that cytokine responses to controlled exercise differed between healthy and illness prone athletes, with athletes prone to URTI showing evidence of impaired inflammatory regulation post-exercise[4]. In the present study, probiotic consumption reduced the magnitude of the difference in acute post-exercise cytokine changes between pre- and post-supplementation in males and females. Subjects on the placebo exhibited a greater increase or decrease (across different cytokines) than those athletes on *L. fermentum (PCC^®^)*. Cytokine expression in individuals infected with respiratory viruses have been proposed as an indicator of disease severity [43] and blocking inflammatory cytokines reduces the severity of disease. A substantial 20-60% reduction in cytokine perturbations associated with probiotic supplementation could represent improved immunoregulatory control. While the cytokine control model remains an attractive explanation for the beneficial effects of probiotics, the experimental evidence linking clinical outcomes with nutritional modulation of immune regulation is lacking.

The increase in total *Lactobacillus *numbers in males is presumably the first step in the mechanism linking supplementation to improved clinical findings in this group. The sample size of the sub-group was adequate to demonstrate the effectiveness of the supplement on gut flora in males. The lack of a substantial difference in total *Lactobacillus *numbers between females on the probiotic and placebo warrants further investigation. In contrast to males on the placebo, females on the placebo had an increase in total *Lactobacillus *numbers in faeces. Retrospective examination of questionnaires addressing yoghurt consumption and dietary practices did not uncover dietary factors to explain the increase. It may be that females would benefit from higher doses of probiotics. The microbiota exerts strong effects on immunity by interacting with a range of receptors on intestinal epithelial cells, M-cells and dendritic cells in the GI tract [44]. The microbiota also enhances immunity beyond the GI tract through interactions with the common mucosal immune system (CMIS) [45]. However, covariate analysis of faecal microbiology and illness symptoms in a subgroup of participants did not reveal a clear relationship that would account for the difference in symptoms between the two groups. This finding is not surprising, given the limited sample size for the bacterial analysis. A much larger sample size is required to fully investigate the role of the microbial flora in mediating the effects on symptoms of illness.

## Conclusion

Supplementation with the probiotic *L. fermentum (PCC^®^) *substantially reduces the severity of self-reported symptoms and illness load of lower respiratory illness, use of cold and flu medication, and severity of gastrointestinal symptoms at higher training loads, in male athletes. These effects may have been mediated via reduction in exercise-induced immune perturbations. The effects on symptoms in females require further investigation. Increased frequency of mild low-grade symptoms of GI illness may reflect short-term adaptive responses in the GI tract with probiotic use. No firm conclusion can be made about the effects of supplementation on URTI.

## Competing interests

DCE and AJ hold full-time positions with Christian Hansen A/S and Probiomics Ltd respectively.

## Authors' contributions

All authors participated in the design of the trial, data interpretation and preparation of the manuscript. AWC, DBP, DCE, NPW and PF were responsible for the design and for the conduct of the project. DBP, AWC and PF oversaw the immunology assays and interpretation of the data. CTC and MAC undertook the faecal analysis and interpretation of the microbiological data. WGH conducted the statistical analyses and modelling of the experimental data. DCE assisted in the interpretation of the data and AJ was responsible for manufacture of the product. All authors read and approved the final manuscript.

## Supplementary Material

Additional file 1 **the Additional material file includes the consort checklist, a more detailed explanation of the analytical approach and the raw values for the cytokines and salivary protein concentrations related to tables 2 through to 6**. Click here for file
