# Supplementary material for: Lactobacillus fermentum (PCC®) supplementation and gastrointestinal and respiratory-tract illness symptoms: a randomised control trial in athletes
Source: Nutr J. 2011 Apr 11;10:30. doi: 10.1186/1475-2891-10-30 (PMC3083335; doi:10.1186/1475-2891-10-30)
Supplement: Additional file 1 — the Additional material file includes the consort checklist, a more detailed explanation of the analytical approach and the raw values for the cytokines and salivary protein concentrations related to tables 2 through to 6. [file 1475-2891-10-30-S1.DOC]

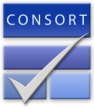
CONSORT 2010 checklist of information to include when reporting a randomised trial*

| Section/Topic | Item No | Checklist item | Reported on page No |
| --- | --- | --- | --- |
| Title and abstract | | | |
|  | 1a | Identification as a randomised trial in the title | 1 |
|  | 1b | Structured summary of trial design, methods, results, and conclusions (for specific guidance see CONSORT for abstracts) | 2 |
| Introduction | | | |
| Background and objectives | 2a | Scientific background and explanation of rationale | 3 |
|  | 2b | Specific objectives or hypotheses | 4 |
| Methods | | | |
| Trial design | 3a | Description of trial design (such as parallel, factorial) including allocation ratio | 4 |
|  | 3b | Important changes to methods after trial commencement (such as eligibility criteria), with reasons | N/A |
| Participants | 4a | Eligibility criteria for participants | 4 |
|  | 4b | Settings and locations where the data were collected | 4 |
| Interventions | 5 | The interventions for each group with sufficient details to allow replication, including how and when they were actually administered | 4 |
| Outcomes | 6a | Completely defined pre-specified primary and secondary outcome measures, including how and when they were assessed | 5-8 |
|  | 6b | Any changes to trial outcomes after the trial commenced, with reasons | N/A |
| Sample size | 7a | How sample size was determined | 8 |
|  | 7b | When applicable, explanation of any interim analyses and stopping guidelines | N/A |
| Randomisation: |  |  |  |
| Sequence generation | 8a | Method used to generate the random allocation sequence | 4 |
|  | 8b | Type of randomisation; details of any restriction (such as blocking and block size) | 4 |
| Allocation concealment mechanism | 9 | Mechanism used to implement the random allocation sequence (such as sequentially numbered containers), describing any steps taken to conceal the sequence until interventions were assigned | N/A |
| Implementation | 10 | Who generated the random allocation sequence, who enrolled participants, and who assigned participants to interventions | 4 |
| Blinding | 11a | If done, who was blinded after assignment to interventions (for example, participants, care providers, those assessing outcomes) and how | 4 |
|  | 11b | If relevant, description of the similarity of interventions | 4 |
| Statistical methods | 12a | Statistical methods used to compare groups for primary and secondary outcomes | 7-8 |
|  | 12b | Methods for additional analyses, such as subgroup analyses and adjusted analyses | 7-8 |
| Results | | | |
| Participant flow (a diagram is strongly recommended) | 13a | For each group, the numbers of participants who were randomly assigned, received intended treatment, and were analysed for the primary outcome | 4 |
|  | 13b | For each group, losses and exclusions after randomisation, together with reasons | 8 |
| Recruitment | 14a | Dates defining the periods of recruitment and follow-up | 4 |
|  | 14b | Why the trial ended or was stopped | N/A |
| Baseline data | 15 | A table showing baseline demographic and clinical characteristics for each group | 16 |
| Numbers analysed | 16 | For each group, number of participants (denominator) included in each analysis and whether the analysis was by original assigned groups | 8 |
| Outcomes and estimation | 17a | For each primary and secondary outcome, results for each group, and the estimated effect size and its precision (such as 95% confidence interval) | 9/10 |
|  | 17b | For binary outcomes, presentation of both absolute and relative effect sizes is recommended | N/A |
| Ancillary analyses | 18 | Results of any other analyses performed, including subgroup analyses and adjusted analyses, distinguishing pre-specified from exploratory | 9/10 |
| Harms | 19 | All important harms or unintended effects in each group (for specific guidance see CONSORT for harms) | 9/10 |
| Discussion | | | |
| Limitations | 20 | Trial limitations, addressing sources of potential bias, imprecision, and, if relevant, multiplicity of analyses | 9-11 |
| Generalisability | 21 | Generalisability (external validity, applicability) of the trial findings | 10-11 |
| Interpretation | 22 | Interpretation consistent with results, balancing benefits and harms, and considering other relevant evidence | 11 |
| Other information | | |  |
| Registration | 23 | Registration number and name of trial registry | N/A |
| Protocol | 24 | Where the full trial protocol can be accessed, if available | N/A |
| Funding | 25 | Sources of funding and other support (such as supply of drugs), role of funders | 1 |

*We strongly recommend reading this statement in conjunction with the CONSORT 2010 Explanation and Elaboration for important clarifications on all the items. If relevant, we also recommend reading CONSORT extensions for cluster randomised trials, non-inferiority and equivalence trials, non-pharmacological treatments, herbal interventions, and pragmatic trials. Additional extensions are forthcoming: for those and for up to date references relevant to this checklist, see [www.consort-statement.org](http://www.consort-statement.org/).

## Analytical approach

Our approach to an inference about the true (large-sample) effect of the supplement on a given symptom was based on the uncertainty in the effect in relation to the smallest clinically important values rather than in relation to the null^(36,37)^. Specifically, we used the same assumptions underlying null-hypothesis testing to derive probabilities that the true effect of the treatment was a substantial increase or decrease in symptoms. An effect with a sufficiently low probability (p) of increasing symptoms (p<0.005 or 0.5%) and a sufficiently high probability of reducing symptoms (p>0.25 or 25%) was deemed to be a clear outcome and reported with the probability of a positive effect interpreted according to the following scale: 0.25-0.75, possible; 0.75-0.95, likely; 0.95-0.995, very likely; >0.995, most likely. A reduction in symptoms with a low probability of occurrence (p<0.25) was deemed to be trivial or negative, depending on the relative probabilities of these two outcomes.  An effect that was possibly positive (p>0.25) but with an unacceptable risk of increasing symptoms (p>0.005) was deemed to be unclear and would require more research with a larger sample before the outcome could be decided. Uncertainty in the effect on a symptom is also shown as the 99% confidence interval, because any overlap of this interval with the smallest clinical value for a substantial increase in symptoms implies that the treatment should not be used. Quantifying the likelihood of both a substantial decrease and/or increase in symptoms in this way is a useful analytical approach in clinically-based research^(22)^ to assist professionals and consumers in making evidence-based choices.

An effect of the probiotic on a bacterial count or on a physiological variable is a mechanistic outcome, so our approach to inference here was based on giving equal importance to substantially positive and negative values. The effect was deemed clear if it was very unlikely (p<0.05) to be substantially positive or very unlikely to be substantially negative, and the outcome was reported according to which magnitude (negative, trivial, positive) had the highest probability. All other effects were deemed unclear. For consistency with this approach, uncertainty in a mechanistic effect is shown as the 90% confidence interval. A given sample size has equal power for clinical and mechanistic inferences^(38)^.

All symptoms were analysed per 100 days. To account for effects of "shoulder" periods following the start and end of supplementation, a proportion of each subject's symptom scores in the shoulder periods was assigned to the subject's accumulated scores for the baseline and supplementation periods. The shoulder periods were assumed to be two weeks, and the proportions of a symptom score in the onset and offset shoulder assigned to the treatment period were d/14 and (14-d)/14 respectively, where d was the count of the days from the start of the shoulder period. The proportions of the score assigned to the baseline and post supplementation period were respectively (14-d)/14 and d/14.

We undertook two kinds of analysis to estimate the effect of the probiotic on symptoms: a comparison of the probiotic- and control-group means only for the period of supplementation (a "post-only" analysis) and a comparison of the group mean changes between baseline and supplementation periods (a "pre-post" analysis). The effects of supplementation on illness symptoms were more precise for the post-only analysis and are the only effects reported here. The number of symptom episodes of a given symptom per 100 days, total number of days of the symptom per 100 days, and total load of the symptom per 100 days (sum of the product of symptom intensity and number of days of the symptom per 100 days) were analysed as ratios: the mean of the probiotic group divided by the mean of the placebo group during treatment. Ratios of 1.20 and 1/1.20 (=0.83) were chosen as smallest clinically important differences. Effects on symptom intensity and on all training-related measures were analysed as differences rather than ratios of means. The smallest important effects for these measures were derived by standardisation: 0.20 of the pooled between-subject standard deviation in the control group and probiotic groups^(39)^. Partly for this reason, descriptive statistics of all measures are presented as mean ± standard deviation. The smallest important effects for these measures were derived by standardisation: 0.20 of the pooled between-subject standard deviation in the control group and probiotic groups^(39)^. Descriptive statistics of all measures are presented as mean ± standard deviation; differences between group means of subject characteristics were assessed with a modification^(36)^ of Cohen's scale^(39)^ for standardised effects (small, 0.20-0.60; moderate, 0.60-1.20; large, >1.20). Confidence limits for the effects on symptom scores and training measures were obtained with bootstrapping. These analyses were performed with the Statistical Analysis System (Version 9.1, SAS Institute, Cary, NC).

Effects of the probiotic on measures of immunology and enteric microflora (Q-PCR data) are presented for pre-post analyses, which were found to give more precision than post-only analyses. These measures were log-transformed before analysis to permit the effect of the treatment to be properly analyzed as factors or percents, and magnitudes of effects were determined by standardisation of the log-transformed variable. The analyses were performed with a spreadsheet^(38)^ that was based on the t statistic for independent samples with unequal variances. Baseline values of the dependent variable were included as a covariate in these analyses to account for regression to the mean. The spreadsheet was also used to investigate the extent to which bacterial counts accounted for symptom scores in this subsample of subjects. In these analyses the log-transformed bacterial count or the pre-post change in the log-transformed count was the covariate, and the dependent variable was rank-transformed.

## Systemic immunity

The effects of supplementation on serum cytokine concentrations at rest are shown in Table 1 (male) and Table 2 (female). Supplementing with *L. fermentum* attenuated reductions in the concentration of several resting serum cytokine concentrations in both males and females.

Table 1 shows that supplementing with *L. fermentum* for 11 weeks attenuated the fall in resting TNF-α concentration in males. TNF-α is a T-helper 1 (Th1) pro-inflammatory cytokine whose primary role is to regulate immune cells and activate inflammatory pathways. Exercise is typically associated with an anti-inflammatory
T-helper 2 (Th2) profile characterised by reductions in Th1 cytokines (IL-6, IFN-γ, GM-CSF and TNF-α). An enhanced Th2 profile has been proposed to increase susceptibility to respiratory infection. There were no substantial differences in the response of the anti-inflammatory or immunoregulatory cytokines to the probiotic treatment in males.

| **Table 1. Changes in resting cytokine concentrations pre and post supplementation in males. Data are shown as a factor change, e.g. a mean difference in the change of means of 1.2 equates to a 20% difference. Units:pg.ml^-1^.** | | | | | | | | |
| --- | --- | --- | --- | --- | --- | --- | --- | --- |
|  | **Probiotic** | |  | **Placebo** | |  | **Difference in change** | |
|  | **Pre-intervention**  **Mean ×⁄÷ SD** | **Post- intervention**  **Mean ×⁄÷ SD** | **% change**  **Mean ± SD** | **Pre-**  **intervention**  **Mean ×⁄÷ SD** | **Post-**  **intervention**  **Mean ×⁄÷ SD** | **% change**  **Mean ± SD** | **Mean;  ×⁄÷ 90%CL** | **Inference** |
| Anti-inflammatory cytokines | | | | | | | | |
| IL-1ra | 114 ×⁄÷ 1.8 | 112 ×⁄÷ 2.2 | -7 **±** 113 | 119 ×⁄÷ 1.8 | 93 ×⁄÷ 2.1 | -22 **±** 73 | 1.19 ×⁄÷1.41 | Inconclusive |
| IL-10 | 5.8 ×⁄÷ 2.8 | 5.9 ×⁄÷ 3.9 | -4 **±** 62 | 5.7 ×⁄÷ 2.4 | 5.0 ×⁄÷ 2.6 | -12 **±** 52 | 1.09 ×⁄÷ 1.26 | Trivial |
| Immuno-regulatory cytokines | | | | | | | | |
| IL-6 | 5 ×⁄÷ 1.8 | 4 ×⁄÷ 2.5 | -16 **±** 117 | 5 ×⁄÷ 1.8 | 4.0 ×⁄÷ 2.5 | -16 **±** 50 | 0.99 ×⁄÷ 1.39 | Inconclusive |
| Pro-inflammatory cytokines | | | | | | | | |
| Il-8 | 2.6 ×⁄÷ 2.5 | 3.2 ×⁄÷ 1.9 | 10 **±** 87 | 2.9 ×⁄÷ 2.1 | 2.9 ×⁄÷ 1.8 | 3.8 **±** 47 | 1.06 ×⁄÷ 1.32 | Inconclusive |
| GM-CSF | 29.6 ×⁄÷ 2.9 | 27.2 ×⁄÷ 4.1 | -17 **±** 166 | 41.8×⁄÷ 3.2 | 27.9×⁄÷ 3.8 | -33 **±** 101 | 1.24 ×⁄÷ 1.56 | Inconclusive |
| IFN-γ | 86 ×⁄÷ 1.6 | 64 ×⁄÷ 2.3 | -27 **±** 113 | 87 ×⁄÷ 2.0 | 55 ×⁄÷ 2.5 | -36 **±** 78 | 1.14 ×⁄÷ 1.42 | Inconclusive |
| TNF-a | 24.0 ×⁄÷ 1.8 | 17.8 ×⁄÷ 2.5 | -30 **±** 80 | 23.0 ×⁄÷ 2.3 | 13.2 ×⁄÷2.4 | -43 **±** 58 | 1.27 ×⁄÷ 1.37 | Smaller reduction |

SD: standard deviation expressed as a times/divide factor of the mean.

90%CL: 90% confidence limits expressed as a times/divide factor of the mean effect

There were substantial differences between females in the probiotic and placebo groups in the change in concentration of IL-8 and GM-CSF cytokines at rest from pre- to post-intervention (Table 2). IL-8 and GM-CSF are pro-inflammatory chemokines. The concentration of both cytokines increased in females taking *L. fermentum,* but decreased in those taking the placebo. This pattern of response is suggestive of an enhanced Th1 profile in females in the probiotic group. There were trivial differences between the two groups in all other cytokines for the female subjects.

| **Table 2. Changes in resting cytokine concentrations pre and post supplementation in females. Data are shown as a factor change, e.g. a mean difference in the change of means of 1.2 equates to a 20% difference. (Units: pg.ml).** | | | | | | | | |
| --- | --- | --- | --- | --- | --- | --- | --- | --- |
| **Cytokines** | **Probiotic** | | | **Placebo** | | | **Difference in change** | |
|  | **Pre**  **Mean ×⁄÷ SD** | **Post**  **Mean ×⁄÷ SD** | **% change**  **Mean ± SD** | **Pre**  **Mean ×⁄÷ SD** | **Post**  **Mean ×⁄÷ SD** | **% change**  **Mean ± SD** | **Mean;  ×⁄÷ 90%CL** | **Inference** |
| Anti-inflammatory cytokines | | | | | | | | |
| IL-1ra | 91.7  ×⁄÷ 2.2 | 90.3  ×⁄÷ 2.0 | -2 ± 68 | 102.3  ×⁄÷ 2.7 | 68.7  ×⁄÷ 1.8 | -21 ± 100 | 1.25  ×⁄÷ 1.53 | Inconclusive |
| IL-10 | 7.2  ×⁄÷ 7.3 | 6.6  ×⁄÷ 6.9 | -8 ± 55 | 4.9  ×⁄÷ 3.1 | 2.9  ×⁄÷ 6.7 | -23 ± 63 | 1.19  ×⁄÷ 1.38 | Trivial |
| Immuno-regulatory cytokine | | | | | | | | |
| IL-6 | 3.1  ×⁄÷ 2.0 | 4.0  ×⁄÷ 1.8 | 6 ± 102.5 | 4.1  ×⁄÷ 3.8 | 2.8  ×⁄÷ 2.5 | -13 ± 165 | 1.21  ×⁄÷ 1.81 | Inconclusive |
| Pro-inflammatory cytokines | | | | | | | | |
| Il-8 | 2.1  ×⁄÷ 2.6 | 2.8  ×⁄÷ 2.3 | 23 ± 53 | 2.7  ×⁄÷ 2.3 | 2.9  ×⁄÷ 2.3 | -3 ± 69 | 1.26  ×⁄÷ 1.40 | Small |
| GM-CSF | 14.7  ×⁄÷ 2.7 | 15.0  ×⁄÷ 3.3 | 2 ± 210 | 32.7  ×⁄÷ 4.8 | 16.0  ×⁄÷ 6.0 | -36 ± 162 | 1.59  ×⁄÷ 2.09 | Small |
| IFN-γ | 70.7  ×⁄÷ 1.6 | 61.3  ×⁄÷ 2.0 | -13 ± 107 | 78.9  ×⁄÷ 3.0 | 47.6  ×⁄÷ 2.5 | -30 ± 128 | 1.23  ×⁄÷ 2.10 | Inconclusive |
| TNF-a | 17.2  ×⁄÷ 2.9 | 14.6  ×⁄÷ 3.5 | -15 ± 95 | 23.2  ×⁄÷ 2.4 | 19.0  ×⁄÷ 2.5 | -13 ± 86 | 0.98  ×⁄÷ 1.6 | Inconclusive |
| SD: standard deviation expressed as a times/divide factor of the mean.  90%CL: 90% confidence limits expressed as a times/divide factor of the mean effect | | | | | | | | |

Table 3 and 4: There were ~20-75% in acute exercise-induced perturbations in cytokines in males and females taking the probiotic following supplementation (Table 3 and Table 4). These tables contain the raw data used to calculate the ratios used in Table 5 in the manuscript.

| **Table 3. Raw cytokine concentrations pre and post VO_2_** **max at baseline and at the end of supplementation in females. Data are shown as a factor change, e.g. a mean difference in the change of means of 1.2 equates to a 20% difference. (Units: pg.ml).** | | | | | | | | | |
| --- | --- | --- | --- | --- | --- | --- | --- | --- | --- |
| **Cytokines** | **Probiotic** | | | | **Placebo** | | |  |  |
|  | **Pre baseline VO_2_**  **Mean ×⁄÷ SD** | **Post baseline**  **VO_2_**  **Mean ×⁄÷ SD** | **Pre**  **End study VO_2_**  **Mean ×⁄÷ SD** | **Post**  **End study**  **VO_2_**  **Mean ×⁄÷ SD** | **Pre baseline VO_2_**  **Mean ×⁄÷ SD** | **Post baseline**  **VO_2_**  **Mean ×⁄÷ SD** | **Pre**  **End study VO_2_**  **Mean ×⁄÷ SD** | **Post**  **End study**  **VO_2_**  **Mean ×⁄÷ SD** | **Inference*** |
| **Anti-inflammatory cytokines** | | | | | | | | | |
| IL-1ra | 91.7  ×⁄÷ 2.2 | 120.0  ×⁄÷ 2.0 | 90.3  ×⁄÷ 2.0 | 91.3  ×⁄÷ 1.7 | 102.3  ×⁄÷ 2.7 | 101  ×⁄÷ 3.0 | 68.7  ×⁄÷ 1.8 | 97.9  ×⁄÷ 1.7 | Very Likely ↓ |
| IL-10 | 7.2  ×⁄÷ 7.3 | 7.7  ×⁄÷ 7.2 | 6.6  ×⁄÷ 6.9 | 6.1  ×⁄÷ 6.9 | 4.9  ×⁄÷ 3.1 | 5.5  ×⁄÷ 3.3 | 2.9  ×⁄÷ 1.8 | 3.9  ×⁄÷ 1.8 | Possible ↓ |
| **Immuno-regulatory cytokine** | | | | | | | | | |
| IL-6 | 3.0  ×⁄÷ 2.0 | 4.4  ×⁄÷ 1.7 | 3.2  ×⁄÷ 2.5 | 3.1  ×⁄÷ 1.9 | 4.1  ×⁄÷ 3.8 | 4.1  ×⁄÷ 4.5 | 2.8  ×⁄÷ 2.5 | 4.0  ×⁄÷ 2.1 | Likely ↓ |
| **Pro-inflammatory cytokines** | | | | | | | | | |
| Il-8 | 2.1  ×⁄÷ 2.6 | 3.2  ×⁄÷ 2.7 | 2.7  ×⁄÷ 2.3 | 2.9  ×⁄÷ 2.2 | 2.7  ×⁄÷ 2.3 | 3.2  ×⁄÷ 2.3 | 2.9  ×⁄÷ 2.3 | 3.3  ×⁄÷ 2.8 | Likely ↓ |
| GM-CSF | 14.7  ×⁄÷ 2.7 | 18.7  ×⁄÷ 3.0 | 15.0  ×⁄÷ 3.3 | 15.5  ×⁄÷ 2.7 | 32.7  ×⁄÷ 4.8 | 23.1  ×⁄÷ 6.5 | 16.0  ×⁄÷ 6.0 | 25.5  ×⁄÷ 3.9 | Very Likely ↓ |
| IFN-γ | 70.7  ×⁄÷ 1.6 | 72.3  ×⁄÷ 2.0 | 61.3  ×⁄÷ 2.0 | 53.9  ×⁄÷ 1.7 | 78.9  ×⁄÷ 3.0 | 85.0  ×⁄÷ 3.1 | 47.6  ×⁄÷ 2.5 | 75.8  ×⁄÷ 2.0 | Likely ↓ |
| TNF-a | 17.2  ×⁄÷ 2.9 | 21.6  ×⁄÷ 2.7 | 14.6  ×⁄÷ 3.5 | 14.6  ×⁄÷ 2.7 | 23.2  ×⁄÷ 2.4 | 15.4  ×⁄÷ 4.2 | 19.0  ×⁄÷ 2.5 | 23.8  ×⁄÷ 2.3 | Very likely ↓ |
| SD: standard deviation expressed as a times/divide factor of the mean.  90%CL: 90% confidence limits expressed as a times/divide factor of the mean effect  *Refer to Table 5 in manuscript | | | | | | | | | |

| **Table 4. Raw cytokine concentrations pre and post VO_2_ at baseline and at the end of supplementation in males. Data are shown as a factor change, e.g. a mean difference in the change of means of 1.2 equates to a 20% difference. (Units: pg.ml).** | | | | | | | | | |
| --- | --- | --- | --- | --- | --- | --- | --- | --- | --- |
| **Cytokines** | **Probiotic** | | | | **Placebo** | | |  |  |
|  | **Pre baseline VO_2_**  **Mean ×⁄÷ SD** | **Post baseline**  **VO_2_**  **Mean ×⁄÷ SD** | **Pre**  **End study VO_2_**  **Mean ×⁄÷ SD** | **Post**  **End study**  **VO_2_**  **Mean ×⁄÷ SD** | **Pre baseline VO_2_**  **Mean ×⁄÷ SD** | **Post baseline**  **VO_2_**  **Mean ×⁄÷ SD** | **Pre**  **End study VO_2_**  **Mean ×⁄÷ SD** | **Post**  **End study**  **VO_2_**  **Mean ×⁄÷ SD** | **Inference*** |
| **Anti-inflammatory cytokines** | | | | | | | | | |
| IL-1ra | 114.4  ×⁄÷ 1.8 | 106.9  ×⁄÷ 2.1 | 111.5  ×⁄÷ 2.2 | 100.6  ×⁄÷ 2.1 | 119.2  ×⁄÷ 1.8 | 107.8  ×⁄÷ 2.2 | 934  ×⁄÷ 2.1 | 114  ×⁄÷ 1.9 | Very Likely ↓ |
| IL-10 | 5.8  ×⁄÷ 2.8 | 5.4  ×⁄÷ 3.2 | 5.9  ×⁄÷ 3.9 | 5.7  ×⁄÷ 3.4 | 5.7  ×⁄÷ 2.4 | 5.5  ×⁄÷ 2.7 | 5.0  ×⁄÷ 2.6 | 5.6  ×⁄÷ 2.6 | Possible ↓ |
| **Immuno-regulatory cytokine** | | | | | | | | | |
| IL-6 | 5.0  ×⁄÷ 1.75 | 4.8  ×⁄÷ 2.0 | 4.2  ×⁄÷ 2.5 | 4.5  ×⁄÷ 2.1 | 4.9  ×⁄÷ 1.8 | 4.2  ×⁄÷ 2.6 | 4.2  ×⁄÷ 2.0 | 4.3  ×⁄÷ 2.6 | Likely ↓ |
| **Pro-inflammatory cytokines** | | | | | | | | | |
| Il-8 | 2.6  ×⁄÷ 2.5 | 3.2  ×⁄÷ 2.3 | 3.2  ×⁄÷ 1.9 | 2.8  ×⁄÷ 2.6 | 2.9  ×⁄÷ 2.1 | 2.8  ×⁄÷ 3.2 | 2.9  ×⁄÷ 1.8 | 2.8  ×⁄÷ 2.6 | Unclear |
| GM-CSF | 29.6  ×⁄÷ 2.9 | 28.3  ×⁄÷ 2.9 | 27.2  ×⁄÷ 4.1 | 26.4  ×⁄÷ 3.8 | 41.8  ×⁄÷ 3.2 | 34.7  ×⁄÷ 2.9 | 27.9  ×⁄÷ 3.8 | 33.2  ×⁄÷ 3.6 | Very Likely ↓ |
| IFN-γ | 86.0  ×⁄÷ 1.6 | 76.9  ×⁄÷ 1.9 | 64.0  ×⁄÷ 2.3 | 71.2  ×⁄÷ 2.1 | 86.8  ×⁄÷ 2.0 | 75.6  ×⁄÷ 2.4 | 55.4  ×⁄÷ 2.5 | 72.0  ×⁄÷ 2.8 | Likely ↓ |
| TNF-a | 23.9  ×⁄÷ 1.8 | 20.1  ×⁄÷ 2.4 | 17.8  ×⁄÷ 2.5 | 18.5  ×⁄÷ 2.2 | 23.0  ×⁄÷ 2.3 | 19.2  ×⁄÷ 2.9 | 13.2  ×⁄÷ 2.4 | 18.3  ×⁄÷ 2.4 | likely ↓ |
| SD: standard deviation expressed as a times/divide factor of the mean.  90%CL: 90% confidence limits expressed as a times/divide factor of the mean effect  *Refer to Table 5 in manuscript | | | | | | | | | |

## Mucosal immunity

The mean changes in the concentrations of salivary proteins are detailed in Table 5 and Table 6. There was a small decrease in the concentration of lactoferrin and small increases in the concentration of SIgA with probiotic treatment in males. There were no substantial differences between treatment groups in the concentration of lysozyme. The salivary proteins were characterised by large variability in protein concentration.

| **Table 5. Concentration of salivary proteins pre and post supplementation, with the effect of the treatment expressed as the difference in the change of the means.** | | | | | | | | |
| --- | --- | --- | --- | --- | --- | --- | --- | --- |
| **Males** | **Probiotic** | |  | **Placebo** | |  | **Effect of treatment** | |
|  | **Pre**  **Mean ×⁄÷ SD** | **Post**  **Mean ×⁄÷ SD** |  | **Pre**  **Mean ×⁄÷ SD** | **Post**  **Mean ×⁄÷ SD** |  | **Mean;  ×⁄÷ 90%CL** | **Inference** |
| Lysozyme | 2200  ×⁄÷ 3.0 | 2500  ×⁄÷ 3.0 |  | 3000  ×⁄÷ 4.4 | 2800  ×⁄÷ 3.1 |  | 0.80; ×⁄÷1.8 | Inconclusive |
| Lysozyme as a ratio to albumin | 53  ×⁄÷ 4.0 | 54  ×⁄÷ 4.0 |  | 87  ×⁄÷ 2.9 | 73 ×⁄÷ 4.6 |  | 0.84; ×⁄÷2.0 | Inconclusive |
| Lysozyme as a ratio to osmolality | 27  ×⁄÷ 2.8 | 31  ×⁄÷ 3.4 |  | 37  ×⁄÷ 2.9 | 31  ×⁄÷ 4.1 |  | 1.08; ×⁄÷2.2 | Inconclusive |
| Lactoferrin | 2900  ×⁄÷ 3.4 | 2700  ×⁄÷ 3.3 |  | 3800  ×⁄÷ 2.2 | 3900  ×⁄÷ 2.6 |  | 0.89; ×⁄÷2.0 | Inconclusive |
| Lactoferrin as a ratio to albumin | 75  ×⁄÷ 2.9 | 57  ×⁄÷ 2.7 |  | 110  ×⁄÷ 2.3 | 96  ×⁄÷ 2.7 |  | 0.69; ×⁄÷1.7 | Small ↓ |
| Lactoferrin as a ratio to osmolality | 37  ×⁄÷ 2.5 | 32  ×⁄÷ 4.0 |  | 43  ×⁄÷ 2.0 | 47  ×⁄÷ 2.5 |  | 0.59; ×⁄÷2.1 | Inconclusive |
| SIgA | 150  ×⁄÷ 2.1 | 170  ×⁄÷ 2.3 |  | 210  ×⁄÷ 1.8 | 200  ×⁄÷ 2.2 |  | 1.39; ×⁄÷1.4 | Small ↑ |
| SIgA as a ratio to albumin | 3.6  ×⁄÷ 2.5 | 3.8  ×⁄÷ 2.3 |  | 6.1  ×⁄÷ 1.8 | 4.8  ×⁄÷ 2.8 |  | 1.34; ×⁄÷1.6 | Small ↑ |
| SIgA as a ratio to osmolality | 1.8  ×⁄÷ 2.0 | 2.0  ×⁄÷ 2.9 |  | 2.5  ×⁄÷ 1.7 | 2.3  ×⁄÷ 2.1 |  | 1.26; ×⁄÷1.6 | Inconclusive |
| SD: standard deviation expressed as a times/divide factor of the mean.  90%CL: 90% confidence limits expressed as a times/divide factor of the mean effect | | | | | | | | |

There were moderate-large decreases in the concentration of lactoferrin with probiotic treatment in females (Table 4). Similar to the males, the concentrations of salivary proteins were characterised by large variability.

| **Table 6. Concentration of salivary proteins pre and post supplementation.** | | | | | | | | |
| --- | --- | --- | --- | --- | --- | --- | --- | --- |
| **Females** | **Probiotic** | |  | **Placebo** | |  | **Difference in change** | |
|  | **Pre**  **Mean ×⁄÷ SD** | **Post**  **Mean ×⁄÷ SD** |  | **Pre**  **Mean ×⁄÷ SD** | **Post**  **Mean ×⁄÷ SD** |  | **Mean;  ×⁄÷ 99%CL** | **Inference** |
| Lysozyme | 2700  ×⁄÷ 6.6 | 3500  ×⁄÷ 4.6 |  | 1700  ×⁄÷ 2.7 | 4300  ×⁄÷ 3.3 |  | 0.51; ×⁄÷2.7 | Inconclusive |
| Lysozyme as a ratio to albumin | 41  ×⁄÷ 3.8 | 100  ×⁄÷ 4.8 |  | 58  ×⁄÷ 2.8 | 75  ×⁄÷ 3.3 |  | 1.69; ×⁄÷2.6 | Inconclusive |
| Lysozyme as a ratio to osmolality | 26  ×⁄÷ 5.2 | 36  ×⁄÷ 3.8 |  | 22  ×⁄÷ 2.5 | 50  ×⁄÷ 2.8 |  | 1.05; ×⁄÷2.5 | Inconclusive |
| Lactoferrin | 4900  ×⁄÷ 3.4 | 4200  ×⁄÷ 2.1 |  | 3500  ×⁄÷ 2.2 | 5700  ×⁄÷ 2.4 |  | 0.44; ×⁄÷2.4 | Moderate ↓ |
| Lactoferrin as a ratio to albumin | 90  ×⁄÷ 1.9 | 130  ×⁄÷ 2.2 |  | 130  ×⁄÷ 3.2 | 100  ×⁄÷ 2.6 |  | 1.15; ×⁄÷3.6 | Inconclusive |
| Lactoferrin as a ratio to osmolality | 50  ×⁄÷ 2.9 | 44  ×⁄÷ 2.3 |  | 40  ×⁄÷ 1.9 | 71  ×⁄÷ 2.5 |  | 0.31; ×⁄÷2.9 | Large ↓ |
| SIgA | 190  ×⁄÷ 2.5 | 160  ×⁄÷ 2.6 |  | 170  ×⁄÷ 1.4 | 180  ×⁄÷ 2.1 |  | 1.00; ×⁄÷2.1 | Inconclusive |
| SIgA as a ratio to albumin | 4.0  ×⁄÷ 3.5 | 4.8  ×⁄÷ 2.2 |  | 6.2  ×⁄÷ 2.4 | 3.4  ×⁄÷ 2.0 |  | 1.98; ×⁄÷2.5 | Inconclusive |
| SIgA as a ratio to osmolality | 2.3  ×⁄÷ 2.4 | 2.1  ×⁄÷ 2.4 |  | 2.0  ×⁄÷ 1.7 | 2.3  ×⁄÷ 2.0 |  | 1.03; ×⁄÷2.4 | Inconclusive |
|  | | | | | | | | |
